# Association between informal help and background factors for persons with multiple sclerosis in Sweden: a cross-sectional study

**DOI:** 10.1136/bmjopen-2024-094418

**Published:** 2025-09-14

**Authors:** Daniel Ståhl, Emilie Friberg

**Affiliations:** 1 Department of Social Work, University of Gothenburg, Gothenburg, Sweden; 2 Department of Clinical Neuroscience, Karolinska Institutet, Stockholm, Sweden

**Keywords:** Multiple sclerosis, Caregivers, Social Support, SOCIAL MEDICINE

## Abstract

**Abstract:**

**Objective:**

Persons living with multiple sclerosis (MS) may experience various symptoms, and some of these persons may need help in daily life. Previous research shows that informal—that is, unpaid—help is more common among persons with MS than formal help. This study aimed to explore the association between socio-demographic background factors and the use of informal help among persons with MS in Sweden, and to investigate any differences in the amount of informal help used per week.

**Design and setting:**

Cross-sectional survey data combined with Swedish register data.

**Participants:**

4412 persons with MS between the ages of 20 and 51 years responded to the survey.

**Outcome measures:**

(1) Use of informal help and (2) the number of hours of informal help used per week. Descriptive statistics and logistic regression and linear regression were used for analysis.

**Results:**

It was found that several socio-demographic background variables were associated with the use of informal help, but a higher degree of impairment, indicated by the Expanded Disability Status Scale for Multiple Sclerosis, had the strongest positive association (OR 6.94, 95% CI 4.70 to 10.03). Fewer variables were associated with the number of hours of informal help per week. The birth country had the strongest positive relationship (B 0.50, 95% CI 0.22 to 0.76) while whether one was living alone had the strongest adverse relationship (B −0.65, 95% CI −0.89 to –0.42). Three of the socio-demographic background variables were found to be positively associated with both the use of informal help and the number of hours of informal help used per week, birth country (OR 1.34, 95% CI 1.00 to 1.79; B 0.50, 95% CI 0.22 to 0.76), receipt of sickness benefit (OR 4.22, 95% CI 3.19 to 5.58; B 0.25, 95% CI 0.05 to 0.44) and higher degree of impairment (OR 6.94, 95% CI 4.70 to 10.03; B 0.31, 95% CI 0.02 to 0.60). Persons with MS born outside the Nordic countries, receiving sickness benefit and severely affected by impairment more often used informal help and they used more hours of informal help per week.

**Conclusions:**

The degree of impairment was the most important factor in determining whether informal help was used. However, the importance of the birth country and receipt of sickness benefit may indicate that subgroups of persons with MS are more vulnerable and referred to informal help rather than formal help.

**STRENGTHS AND LIMITATIONS OF THIS STUDY**

- This survey was sent to everyone 20–50 in the national Swedish multiple sclerosis register.
- The combination of data from three sources made it possible to examine the importance of a range of clinical and socio-demographic background factors.
- The cross-sectional nature of the study limits the interpretation of the direction of the relationships.
- The degree of informal help received among non-responders is unknown.

## Introduction

 The prevalence of persons living with multiple sclerosis (MS) worldwide has increased over the course of measurements made in 2008, 2013 and 2020.^1^ The disease is more common in Europe and North America than the rest of the world, and women are more often affected by MS than men.^1 2^ Sweden has a high prevalence of persons with MS.^3^ Many persons diagnosed with MS are between the ages of 20 and 40 years.^2^ MS is heterogeneous and unpredictable in character. The disease may lead to several symptoms such as fatigue, depression, cognitive impairment, impaired vision, sensory symptoms, sexual dysfunction, impaired bladder function and impaired movement.^2 4^ Many of these symptoms are invisible to others. Invisible symptoms seem to be prevalent among many persons with MS, even those that may appear to be mildly affected by the disease.^4^ Many of the symptoms mentioned may involve a need for help in everyday life. Help that is performed by a paid employee (eg, personal assistant) is defined as formal help. Help performed by someone who does not receive any payment (eg, spouse or child) is defined as informal help.^5^

Previous research on formal and informal help among persons living with different chronic conditions, including MS, found that the majority of the help used was informal.^5 6^ Informal help was mainly performed by a spouse/partner, followed by a child.^6^ This pattern is the same in studies that specifically focus on persons with MS from various national contexts,^7^,^11^ and the role of informal helpers is mostly occupied by women.^7 11^ One study involving persons with MS from several European countries found that around 46% of respondents used informal help from family members.^8^ This figure is broadly consistent with other estimates, although data on this is lacking from various national contexts.^12^ A smaller study from Greece reported that around 58% of respondents used informal help.^13^ In a Swedish study that primarily examined the societal costs related to both formal and informal help, it was found that 49% of the respondents used informal help to some extent.^14^

Previous studies report various numbers of hours of informal help that persons with MS use per week, depending on their samples; older and more severely affected persons tend to use more hours of informal help (eg, ^6 10 11^). The degree of impairment in persons with MS is commonly measured according to the EDSS (Expanded Disability Status Scale in Multiple Sclerosis).^2^ Few studies have applied this measure in relation to informal help. However, an older study found that, in certain cases, persons with MS who were classified as moderately or severely disabled did not use any form of formal help.^15^ Subsequent studies have found an association between a higher degree of impairment according to the EDSS and the use of informal help.^9 13^ MS can affect a person with a variety of symptoms, many of them invisible to others. Patten *et al*^16^ found that persons with MS reported unmet needs in daily living more often than those with other chronic conditions. Fatigue is a common invisible symptom that many persons with MS experience. A previous study found that being affected by fatigue was associated with a higher likelihood of using informal help than formal help. This pattern was also evident for those with higher EDSS scores, indicating moderate and severe impairment.^17^

Previous research on informal help in general in Sweden has mainly focused on help provided to older persons.^18^ The Swedish universal welfare system has been contested, as several studies have found that formal help is decreasing and informal help is increasing (eg, 19,21). Scholars have used the theoretical concepts of de-universalisation/re-familialisation to describe a process in which more responsibilities for helping older persons are shifted from public to family and relatives.^19 22^ The informal helpers of older persons in Sweden are mainly female. Female helpers perform more hours of informal help than men, and they are more often the sole informal helpers.^20 23^ Informal helpers are most often a spouse/partner, followed by children.^19 24^ Dahlberg *et al*^19^ found that women use formal help more often, while men use informal help more often, in line with women’s generally longer life span. However, living alone was the most influential background factor associated with the use of formal help and vice versa for informal help among older persons.^19^ A previous study found that austerity measures in Sweden have created new forms of inequality among older persons. Those with lower education tend to be more dependent on informal help from family and relatives, while those with higher education and thus better socioeconomic conditions, can purchase these services from market providers.^21^ Mac Innes^25^ suggested that being born in the country of residence or being from a nation with a similar type of welfare state entails better capacity to use available formal help. Wimo *et al*^24^ found differences in the use of formal and informal help among older persons living in small municipalities, medium-sized municipalities and urban areas. Older persons in small municipalities used only informal help to a greater extent than those in mid-sized municipalities and urban areas. The time of informal help use was higher than in the other two cases. The use of formal help was, on the other hand, more common in urban areas.^24^

To summarise, the state of knowledge about persons with MS and informal help is limited. This is because many studies are based on relatively small samples (eg, ^13^), samples with a relatively high average age (eg, ^10^) and/or a high degree of impairment (eg, ^14^). There are few Swedish studies in this area, despite the described development towards de-universalisation. Previous research has shown that various socio-demographic background factors can influence whether persons in need of help due to disease or age use informal help. Thus, the aim of this study was to explore the association between socio-demographic background factors and the use of informal help among persons with MS in Sweden. Furthermore, the aim was to investigate any differences in the amount of informal help used in relation to the background factors.

## Methods

This study was based on a cross-sectional survey of various aspects of everyday life related to MS, distributed in May 2021 to all between the ages of 20 and 50 years listed in the Swedish MS Registry.^26^ A total of 8458 persons with MS were invited to participate in a web-based questionnaire administered by Statistics Sweden. The response rate was 52% (4412 participants) after up to four reminders. Among those who did not respond, a higher proportion were younger, male, were born outside Sweden and had a lower income than those who responded. Informed consent was obtained from all participants during the survey.

### Patient and public involvement statement

The cross-sectional survey on which the study is based was developed based on literature and input from researchers, clinicians and representatives from Ung med MS (English: young persons with MS), a non-profit organisation targeting young persons living with MS.

### Data

In addition to the survey, data were obtained from Statistics Sweden and the Swedish MS Registry. The survey asked for information on several aspects of life. Respondents were asked whether they used informal help, but the question used the more colloquial term unpaid help and was worded as follows: ‘Do you receive any unpaid support/help from someone close to you because of your MS? (The support can be both at home and in relation to work/studies)’. If yes, respondents were further asked how many hours of informal help they used per week and who helped them. Furthermore, the survey contained questions about which MS symptoms currently affected daily life the most, whether they had another long-term disease/impairment, the occupation of the respondent and whether the respondent was cohabitating, among other things. Data on sex, age, birth country, level of education, type of residential area and disposable income in 2019 were derived from Statistics Sweden’s Longitudinal Integrated Database for Health Insurance and Labor Market Studies.^27^ Data on the respondents’ most recent EDSS scores were obtained from the Swedish MS Registry.^26^ In cases where the most recent EDSS score was 3 years old or older, the data were considered missing. Statistics Sweden linked the data sources using Swedish personal identification numbers, performed the non-responder analyses and delivered anonymised data to the researchers.

### Dependent and independent variables

This study used two dependent variables based on self-reported responses: the binary categorical variable ‘use of informal help’ (reference group no) and the continuous variable ‘number of hours of informal help used per week’. The number of hours of informal help used per week was skewed (5.80). To meet the assumptions of linear regression, the variable was transformed using the natural logarithm (skewness 0.06).^28^

Several socio-demographic background variables were used as independent variables: sex (women, men), age (20–30, 31–40, 41–51), educational level (0–12 years in school, >12 years in school), birth country (Nordic countries including Sweden, other countries), cohabitation (no, yes), reception of sickness benefit (no, yes), type of living area (living in a city, town/suburban area, rural area), disposable income (≤165 240 Swedish kronor, >165 240 Swedish kronor), most limiting symptom of MS (invisible symptom, visible symptom, no symptom), EDSS score (0–2.5, 3–5.5, 6–9.5) and presence of another long-term disease/impairment (no, yes). Disposable income from 2019 was heavily skewed (38.02) and therefore divided into a binary variable (≤165 240 Swedish kronor, >165 240 Swedish kronor). This categorisation was based on the Eurostat risk of poverty measure.^29^ The categorisation of the EDSS variable was based on clinical thresholds,^30^ where the group between 0 and 2.5 corresponds to mild MS, 3–5.5 corresponds to moderate MS and 6–9.5 corresponds to severe MS. The variable reception of sickness benefit included sick leave compensation and/or disability pensions. Respondents were asked to respond to the question, ‘Which MS symptom do you find the most limiting?’. As this was an open-ended question, the responses were first grouped based on commonly employed categorisations.^31 32^ Second, the responses were coded into the following outcomes: invisible symptoms (eg, fatigue), visible symptoms (eg, impaired mobility) and no symptoms.^33^ For logistic regression analysis, this variable was recoded by using the missing indicator method^34^ by making missing responses an outcome. This was done to avoid losing too many respondents to the mutually adjusted logistic regression model. For the multivariate linear regression analysis, this variable was recoded into a binary variable, including only invisible and visible symptoms, due to the low number of responses indicating no symptoms. The EDSS score was included to measure degree of impairment. A higher score on this scale indicates more severe impairment.^35^ The variable was recoded for logistic regression analysis in a similar way to the variable of most limiting symptoms, using the missing indicator method.^34^

### Statistical analysis

Descriptive analyses are shown in table 1, including numbers and percentages of persons with MS using or not using informal help plus median values for the number of hours of informal help used per week. Before testing the independent variables in the logistic regression, they were analysed using the dependent variable in cross-tabulations. Both χ^2^ tests and Fisher’s exact tests were performed because of the assumptions of goodness-of-fit tests of logistic regression.^36^ A test to control for potential multicollinearity between independent variables confirmed that no multicollinearity was found^37^ (see online supplemental table 1). Several potential interaction effects were examined, and one statistically significant interaction was found: educational level×receipt of sickness benefit. All independent variables were used separately in a logistic regression analysis to obtain unadjusted ORs. In the next step, all independent variables were simultaneously used in logistic regression analysis, which also included the interaction between educational level and receipt of sickness benefit (n=4322). Dummy variables corresponding to the same reference categories used in the logistic regression analysis were coded for use in the linear regression analysis (n=515). Potential multicollinearity between the independent variables was also inspected using multivariate linear regression analysis. No multicollinearity was found^37^ (see online supplemental table 2). The natural logarithm of the number of hours of informal help used per week expressed a linear relationship with the independent variables^38^ (see online supplemental figure 1), and the residuals did not show a systematic pattern, such as a bow^37^ (see online supplemental figure 2). The alpha level was set to ≤0.05 for statistical significance. All analyses were performed using IBM SPSS Statistics V.28.0 (IBM, Armonk, New York, USA).

**Table 1:** Descriptive statistics of persons with MS responding to the question about use of informal help and median values of responses to number of hours of informal help used per week

	Not using informal help n=3740	Using informal help n=636	Number of hours of informal help used per week - median (IQR) n=520
Sex n (%)			
Female	2645 (70.7)	476 (74.8)	5 (3–10)
Male	1095 (29.3)	160 (25.2)	5 (2–10)
Age n (%)			
20–30 years	444 (11.9)	77 (12.1)	5 (2–12)
31–40 years	1312 (35.1)	233 (36.6)	5 (2–10)
41–51 years	1984 (53)	326 (51.3)	6 (3–12)
mean (SD)	40 (7.2)	39.9 (7.4)	
Educational level (years in school) n (%)			
0–12	1422 (38)	301 (47.3)	5 (3–10)
>12	2318 (62)	335 (52.7)	7 (3–12)
Birth country n (%)			
Nordic countries	3369 (90.1)	551 (86.6)	5 (2–10)
Outside Nordic countries	371 (9.9)	85 (13.4)	10 (5–20)
Cohabitation n (%)			
Living by oneself	549 (14.7)	116 (18.3)	7 (4–11)
Cohabiting with adult and/or child(ren)	3189 (85.3)	519 (81.7)	3 (1–6)
Reception of sickness benefit n (%)			
Yes	584 (15.6)	321 (50.5)	5 (2–10)
No	3156 (84.4)	315 (49.5)	7 (4–14)
Place of residence n (%)			
City	1678 (44.9)	268 (42.1)	5 (2–10)
Town/suburb	1443 (38.6)	247 (38.8)	6 (4–12)
Rural area	613 (16.4)	121 (19)	6 (3–10)
Disposable income 2019 n (%)			
<SEK 165 240 per year	533 (14.3)	198 (31.2)	5 (2–10)
>SEK 165 240 per year	3200 (85.7)	437 (68.8)	7 (3–12)
Mean (SD)	313 479 (330–672)	255 168 (256–369)	
Most limiting symptom of MS n (%)			
Invisible symptom	2289 (61.2)	457 (73.8)	6 (3–10)
Visible symptom	614 (16.4)	154 (24.9)	5 (2–10)
No symptom	523 (14)	8 (1.3)	2 (1–2)
Missing	314 (8.4)	17 (2.7)	2 (1–5)
EDSS n (%)			
0–2.5	2557 (68.4)	251 (39.5)	5 (2–10)
3–5.5	360 (9.6)	147 (23.1)	5 (2–10)
6–9.5	74 (2)	98 (15.4)	8 (4–15)
Missing	749 (20)	140 (22)	7 (4–15)
Presence of another long-term disease/impairment n (%)			
Yes	1096 (29.6)	259 (41.2)	5 (3–10)
No	2608 (70.4)	369 (58.8)	6 (3–12)

EDSS, Expanded Disability Status Scale for Multiple Sclerosis; MS, multiple sclerosis; SEK, Swedish kronor.

## Results

### Descriptive results

Respondents’ characteristics are presented in table 1. The age range is between 20 and 51 years, which is due to the fact that some of the respondents turned 51 before answering the questionnaire. About one-fifth of the sample used informal help, and there were several variables for which persons who used informal help differed from those who did not. Persons with MS who used informal help were generally less educated, born outside the Nordic countries, received sickness benefit(s), had less disposable income, were more often affected by symptoms of MS (visible or invisible), had a higher EDSS score and had another long-term disease/impairment. Table 1 also shows the median values for persons with MS responding to the number of hours of informal help used per week in relation to background factors. The background factor with the largest discrepancy in median values between outcomes was country of birth. Responses to the survey question, ‘Who is helping you?’ were distributed in the following order: ‘spouse/partner’ 61.35%, ‘parents or siblings’ 45.19%, ‘children’ 21.92% and ‘other relative’ 14.62%. Note that respondents were able to choose one or several responses.

### Use of informal help

In the mutually adjusted logistic regression model, 8 of the 11 variables remained statistically significant (see table 2 for full stats). The most tangible association with use of informal help was an EDSS score between 6 and 9.5, followed by receipt of sickness benefit. Two of the statistically significant variables and outcomes were related to lower ORs of using informal help, being between 41–51 years of age and having reported not being affected by any MS symptom. Variables that were not found to be statistically significantly associated with the use of informal help were sex, place of residence and whether one was living alone. The statistically significant interaction effect found between reception of sickness benefit and educational level (see Methods) was included in the model. This interaction indicates that the positive association between receiving sickness benefit and use of informal help is weaker among those with higher education.

**Table 2:** Unadjusted and adjusted ORs from binary logistic regression analyses for characteristics associated with usage of informal help among persons with multiple sclerosis, n=4322

	Unadjusted		Adjusted	
	OR	95% CI	OR	95% CI
Sex				
Male	0.79*	0.65 to 0.96	0.91	0.73 to 1.14
(Ref=female)				
Age				
31–40	1.02	0.77 to 1.35	0.88	0.65 to 1.20
41–51	0.94	0.72 to 1.24	0.57***	0.42 to 0.78
(Ref=20–30)				
Educational level				
0–12 years	1.46***	1.23 to 1.73	1.33*	1.03 to 1.71
(Ref=>12 years)				
Birth country				
Outside Nordic countries	1.43**	1.10 to 1.83	1.34*	1.00, 1.79
(Ref=Nordic countries, incl. Sweden)				
Place of residence				
Towns and suburbs	1.06	0.88 to 1.28	1.00	0.81, 1.23
Rural areas	1.24	0.98 to 1.56	1.08	0.83 to 1.41
(Ref=cities)				
Cohabitation				
Living by oneself	1.32*	1.05 to 1.64	0.95	0.74 to 1.23
(Ref=cohabitating)				
Disposable income				
<SEK 165 240 per year	2.72***	2.24 to 3.30	1.47**	1.17 to 1.85
(Ref=>SEK 165 240 per year)				
Reception of sickness benefit				
Yes	5.58***	4.66 to 6.68	4.22***	3.19 to 5.58
(Ref=no)				
EDSS				
3–5.5	4.20***	3.33 to 5.30	2.56***	1.98 to 3.31
6–9.5	13.60***	9.79 to 19.10	6.94***	4.70 to 10.03
(Ref=0–2.5)				
Presence of another long-term disease/impairment				
Yes	1.67***	1.40 to 1.99	1.25*	1.02 to 1.51
(Ref=no)				
Most limiting symptom				
Visible symptom	1.27*	1.03 to 1.55	1.08	0.85 to 1.36
No symptom	0.14***	0.09 to 0.21	0.22***	0.14 to 0.33
(Ref=invisible symptom)				
Reception of sickness benefit×educational level	0.59**	0.41 to 0.85	0.52**	0.36 to 0.77

Model: Nagelkerke R2 0.234, Hosmer and Lemeshow test 0.732.

*p<0.05, **p<0.01, ***p<0.001.

EDSS, Expanded Disability Status Scale for Multiple Sclerosis; SEK, Swedish kronor.

### Number of hours of informal help used per week

The natural logarithm of responses to the number of hours of informal help used per week was analysed using the same background factors as the previously described model (see table 3 for full stats). 5 of the 11 variables were statistically significant. The variable with the greatest impact was cohabitation. Persons with MS living alone used a significantly smaller number of hours of informal help per week. Respondents born outside the Nordic countries were found to use a significantly greater number of hours of informal help per week than those born in the Nordic countries. The number of hours of informal help per week decreased for persons with MS in the age range of 31–40 compared with the youngest group, ages 20–30. It was found that respondents receiving sickness benefit used more informal help per week than those who did not receive it. Lastly, those with an EDSS score between 6 and 9.5 used significantly more hours of informal help per week. The adjusted R^2^ value was 0.131, meaning that the independent variables were only able to explain 13.1% of the variance of the dependent variable.

**Table 3:** Linear model of background factors associated with the natural logarithm of number of hours of informal help per week used by persons with multiple sclerosis, n=515

	B (95% CI)	SE	Beta	T value
(Constant)	1.1 (0.40 to 1.80)	0.36		3.09
Sex				
Male	0.03 (−0.13 to 0.30)	0.11	0.09	0.79
(Ref=female)				
Age				
31–40	−0.33* (−0.65 to −0.22)	0.16	−0.15	−2.10
41–51	−0.10 (−0.42 to 0.21)	0.16	−0.05	−0.68
(Ref=20–30)				
Educational level				
0–12 years	0.08 (−0.11 to 0.26)	0.09	0.04	0.80
(Ref=>12 years)				
Birth country				
Outside Nordic countries	0.50*** (0.22 to 0.76)	0.14	0.15	3.55
(Ref=Nordic countries, incl. Sweden)				
Place of residence				
Towns and suburbs	0.16 (−0.04 to 0.36)	0.10	0.08	1.61
Rural areas	0.06 (−0.21 to 0.30)	0.13	0.02	0.35
(Ref=cities)				
Cohabitation				
Living by oneself	−0.65*** (−0.89 to −0.42)	0.12	−0.24	−5.47
(Ref=cohabitating)				
Disposable income				
<SEK 165 240 per year	0.17 (−0.04 to 0.37)	0.10	0.07	1.59
(Ref=>SEK 165 240 per year)				
Reception of sickness benefit				
Yes	0.25* (0.05 to 0.44)	0.10	0.012	2.51
(Ref=no)				
EDSS				
3–5.5	−0.03 (−0.27 to 0.20)	0.12	−0.01	−0.28
6–9.5	0.31* (0.02 to 0.60)	0.15	0.11	2.08
(Ref=0–2.5)				
Presence of another long-term disease/impairment				
Yes	0.09 (−0.09 to 0.27)	0.09	0.04	1.02
(Ref=no)				
Most limiting symptom				
Visible symptom	−0.12 (−0.33 to 0.10)	0.11	−0.05	−1.07
(Ref=invisible symptom)				

Model: R2 0.158, adjusted R2 0.131.

*p<0.05, **p<0.01, ***p<0.001.

EDSS, Expanded Disability Status Scale for Multiple Sclerosis; SEK, Swedish kronor.

## Discussion

The central finding of this study is that birth country, receipt of sickness benefit and an EDSS score between 6 and 9.5 was statistically significantly associated with both the use of informal help and a positive relationship with the number of hours of informal help used per week. Persons with MS born outside Nordic countries used informal help to a greater extent than those born within Nordic countries, and the difference in median values for the number of hours of informal help per week was 5 hours. This finding is worth highlighting in relation to a previous study on formal help for persons with MS, as birth country was not found to be statistically significantly associated with either use of personal assistance or home help.^39^ It has been proposed that persons born in nations with different welfare systems may face difficulties due to language barriers and less knowledge about Swedish welfare benefits. Age at migration is an important factor, as older age increases potential difficulties.^25^ There is limited knowledge on this topic in Sweden, but this study supports these difficulties.

The impact of receiving sickness benefit may appear surprising at first sight. However, it needs to be emphasised that the variable with the greatest influence on the use of informal help was the EDSS score, suggesting that the degree of impairment is the most important factor behind the use of informal help. Persons with MS who have a higher degree of impairment more often receive some form of sickness benefit.^8 40^ The EDSS score has previously been found to be the most significant factor associated with formal help for persons with MS^39^ and was found to be highly influential in this study as well.

This study found that the majority of the background factors studied were statistically significantly associated with the use of informal help. The OR for the use of informal help was lower for persons with MS in the oldest age group than for those in the youngest group. On the one hand, this finding was somewhat surprising considering the association between higher age and impairment.^41^ On the other hand, the degree of impairment, rather than age itself, is a factor associated with higher use of both informal^13^ and formal help,^39^ and MS is a heterogeneous disease with different manifestations and courses.^2 12^ Persons with MS in the older group were typically more likely to have an EDSS score above 2.5 than those in the youngest group. A higher EDSS score has previously been found to be associated with use of formal help.^39^ The findings regarding use of informal help from the present study partially conform to previous research, for example, regarding lower educational level,^21^ being born outside Nordic countries^25^ and higher EDSS scores.^9 13^ However, no significant differences were found in this study considering sex or whether one was living alone (cf. ^19^), place of residence (cf. ^24^) or visible symptoms in relation to invisible symptoms (cf. ^17^).

Fewer variables were statistically significantly associated with the number of hours of informal help used per week. The number of hours of informal help used per week was found to decrease for those aged 31–40 in comparison to the youngest group as well as for those living alone. These findings are interesting to compare to previous research, as increasing age has been shown to increase the amount of informal help (eg, ^6 10 11^). A plausible explanation for this difference might be the difference in welfare contexts (cf. ^42^), as the cited studies are from North America. However, the difference needs to be interpreted with caution, as the samples in previous studies were both older and more affected by impairment.^8 14^ A strong negative relationship between the number of hours of informal help and living alone was expected, as this help is mostly provided by a spouse or partner (eg, ^6 7 14^). The latter was supported by the respondents of this study. The number of hours of informal help used per week increased for respondents who received sickness benefit, were born outside Nordic countries and those with an EDSS score between 6 and 9.5. While the findings considering reception of sickness benefit and higher degree of impairment may be considered to match expectations (cf. ^9 40^), the finding regarding birth country is more surprising considering the strong association. As previously discussed, differences in knowledge about available formal help within the welfare system^25^ might explain the differences in its use. However, less is known from the literature about tangible differences in the number of hours per week.

In the present sample, 14.42% (n=636) used informal help. This figure is considerably lower than reported in previous studies.^8 14^ This difference can probably be explained by the comparatively young and not (yet?) particularly impaired sample in this study. Despite the relatively low proportion of users of informal help in the sample, the proportion of formal help users was significantly lower,^39^ in line with other studies (eg, ^6 7 9^). There were also some differences between the results of the logistic regression and the linear regression, worthy of attention, for example, regarding age. The logistic regression analysis showed a statistically significant lower OR for the use of informal help for the group aged 41–51 compared with the reference group, while the linear regression analysis showed a statistically significant negative relationship with the number of hours of informal help used per week for the 31–40 age group compared with the reference group. Another interesting difference was that the cohabitation variable was only statistically significant in the linear regression analysis, with an expected negative relationship (cf. ^6 7 19^) to the dependent variable.

The combined findings of the present study and the previous one, focusing on formal help,^39^ imply that the usage of help, regardless of whether it is in an informal or formal arrangement, primarily depends on the degree of impairment. On a descriptive level, it was found that persons with MS using formal help generally had higher EDSS scores^39^ than those using informal help. This can be understood to be in line with the objective of the universal welfare state where the degree of impairment should determine who is granted formal help.^22^ As the sample used in the present study was both generally younger and less impaired than samples from previous research (cf. ^6 10 11^), it can be assumed that persons with MS generally initiate informal help when first in need of help rather than seeking formal help. However, this study found several associations between background factors corresponding to lower socioeconomic status and informal help. For example, lower educational level, receipt of sickness benefit and disposable income below Swedish kronor 165 240 per year. Furthermore, other variables that were found to be statistically significant are known to be risk factors for being socioeconomically vulnerable, such as being born outside Nordic countries,^25^ a higher degree of impairment^40 43^ and having another long-term disease/impairment.^44^ It has been argued that de-universalisation increases inequality in society,^45^ and the result may possibly speak for it.

Another important point to raise, which is related to disability policy, is that for a large proportion of persons with MS the impact of the disease is invisible^4^ and/or fluctuating.^46^ The often invisible impact of the disease was also reflected in this study. In terms of the proportion of persons with MS who used informal help, those who reported an invisible symptom as the most limiting accounted for the substantially largest group. In our previous study, we found that visible symptoms were related to the use of personal assistance, while invisible symptoms were related to the less extensive help form of home help services.^39^ As impairment is often perceived as something visible and stable, in the sense of constant,^46 47^ persons with MS risk being perceived as less affected by impairment than they often are in reality.^4^ This could help explain the consistent finding that informal help is more widely used than formal help among persons with MS. This suggests that invisible symptoms need to be given greater attention within healthcare and by the authorities that decide on formal help.

### Suggestions for future research

This study can suggest several directions for future research. In line with what was discussed in the previous paragraph, we suggest that future studies further investigate the impact of invisible and fluctuating symptoms of MS. Although we included the presence of comorbidity, future studies could explore the issue in more depth by, for example, more specifically analysing possible differences between types of diseases/impairments. The low adjusted R^2^ value obtained from the linear regression analysis indicates that future studies should further investigate which variables have an impact on the amount of informal help used among persons with MS. It is suggested that future research should further explore differences in the use of informal help between persons with MS who are domestically born and those born in countries with other types of welfare systems. Furthermore, there is a need for longitudinal studies that would be able to outline any changes in variables associated with the use of informal help over time, but also contribute knowledge on any plausible changes in line with de-universalisation.^22^ It can also be suggested that qualitative research might be able to explore how persons with MS experience and relate to a situation where they are in need of help. Is the degree of impairment the most influential factor when persons with MS themselves describe their path towards seeking help?

### Strengths and limitations

This article contributes towards filling an identified knowledge gap.^12 18^ The combination of data from the three sources provided an abundant substrate for persons with MS and enabled an exploration of the importance of a variety of both clinical and socio-demographic background factors. The sample provides results for an extensive share of persons with MS in Sweden (cf. ^14^), and studies on younger persons with MS and informal help are scarce (cf. ^10^). Causality cannot be demonstrated based on cross-sectional data, and the direction of the associations have to be assumed based on what is known from previous research.^37^

It must be acknowledged that this study is also subject to some limitations. The proportions of non-respondents were higher in certain groups, which is common in survey-based research (eg, 48,50) (see online supplemental table 3). This may have been due to a number of reasons, such as the survey being too extensive, the content being perceived as irrelevant^48^ or language being a barrier for those born in other countries.^48 49^ It is conceivable that these factors may have influenced the response rate among those invited to participate. Another factor that may be involved is sociocultural differences in what is perceived to be the responsibility of relatives in helping a sick loved one (cf. ^13^). Such differences could be based on whether the persons were born in a country with a less general welfare system than Sweden (cf. ^25^). We cannot know the extent of use of informal help among non-responders, but the interpretation of the extent of informal help in these groups should be made with caution.

The sample consisted of persons with MS between 20 and 51 years old, due to being collected primarily to focus on younger persons with MS of working age. On the one hand, the young sample is a strength, as previous studies are often based on older samples (eg, ^10^). On the other hand, inclusion of older persons with MS would probably have led to a greater discrepancy between impairment levels within the sample. The survey contained a question about who provides informal help for persons with MS, and it confirmed that the case of Sweden was similar to other countries. However, the survey did not include any requests for more specific information about the helper, whether it was a woman or man.

Lastly, various variables were recoded before being included in the analyses. For example, birth country was coded as a binary variable based on the fact that a large majority of respondents were born in Sweden. Because of this, several other outcomes (eg, Africa) included very few observations. Based on the fact that the Nordic countries are usually classified as universal welfare states,^42^ these countries were therefore merged and compared against the others. Statistically, these measures were necessary. However, these actions meant that it was not always possible to reflect the complex reality of the respondents’ situation.

## Conclusions

The present study found that several of the socio-demographic background variables were associated with the use of informal help for persons with MS. A higher degree of impairment, as measured by EDSS, had the strongest relationship with the use of informal help. Fewer variables were associated with the number of hours of informal help per week. The birth country and whether one was living alone were the most significant background factors. Three variables were found to be associated with both the use of informal help and the number of hours of informal help used per week: birth country, receipt of sickness benefit and EDSS. Persons with MS born outside Nordic countries more often used informal help, and they used more hours of informal help per week. The pattern was the same for persons with MS who received any form of sickness benefit and had an EDSS score between 6 and 9.5. The results indicate that the degree of impairment is the most significant factor for using or not using informal help. However, the importance of birth country and receipt of sickness benefit may be a sign that subgroups of persons with MS are more vulnerable and referred to informal help rather than formal help. Suggestions for future research are proposed to better understand whether inequality is increasing with the likely ongoing de-universalisation of the welfare system. This, in turn, can make an important contribution to the future development of disability policy.

## Supplementary material

10.1136/bmjopen-2024-094418 online supplemental file 1

10.1136/bmjopen-2024-094418 online supplemental file 2

10.1136/bmjopen-2024-094418 online supplemental file 3

## Data Availability

No data are available.
