# Supplementary material for: Association between informal help and background factors for persons with multiple sclerosis in Sweden: a cross-sectional study
Source: BMJ Open. 2025 Sep 14;15(9):e094418. doi: 10.1136/bmjopen-2024-094418 (PMC12434731; doi:10.1136/bmjopen-2024-094418)
Supplement: online supplemental file 1 [file bmjopen-15-9-s001.docx]

**Supplementary file 1 – Multicollinearity analysis**

**Supplementary table 1 - Multicollinearity analysis for the mutually adjusted logistic regression model**

| Variable | VIF | Tolerance |
| --- | --- | --- |
| Sex | 1,089 | 0,919 |
| Age | 1,228 | 0,814 |
| Educational level | 1,826 | 0,548 |
| Birth country | 1,062 | 0,942 |
| Place of residence | 1,117 | 0,895 |
| Cohabitation | 1,077 | 0,928 |
| Disposable income | 1,178 | 0,849 |
| Reception of sickness benefit | 2,176 | 0,460 |
| EDSS | 1,342 | 0,745 |
| Presence of another long-term disease/impairment | 1,052 | 0,951 |
| Most limiting symptom | 1,130 | 0,885 |
| Receipt of sickness benefit x Educational level | 2,841 | 0,352 |

**Supplementary table 2 - Multicollinearity analysis for the linear regression model**

| Variable | VIF | Tolerance |
| --- | --- | --- |
| Sex | 1,120 | 0,893 |
| Age | 1,224 | 0,817 |
| Educational level | 1,132 | 0,884 |
| Birth country | 1,064 | 0,940 |
| Place of residence | 1,136 | 0,880 |
| Cohabitation | 1,124 | 0,890 |
| Disposable income | 1,162 | 0,860 |
| Reception of sickness benefit | 1,250 | 0,800 |
| EDSS | 1,370 | 0,730 |
| Presence of another long-term disease/impairment | 1,065 | 0,939 |
| Most limiting symptom | 1,144 | 0,874 |
