# Supplementary material for: Association between informal help and background factors for persons with multiple sclerosis in Sweden: a cross-sectional study
Source: BMJ Open. 2025 Sep 14;15(9):e094418. doi: 10.1136/bmjopen-2024-094418 (PMC12434731; doi:10.1136/bmjopen-2024-094418)

**Supplementary file 2 – Examination of linear regression assumptions**

**Supplementary figure 1 - PP Plot examination of linearity between independent variables and the dependent variable in linear model of background factors associated with the natural logarithm of number of hours of informal help per week used by persons with MS**

**Supplementary figure 2 - Residual plot of linear model of background factors associated with the natural logarithm of number of hours of informal help per week used by persons with MS**


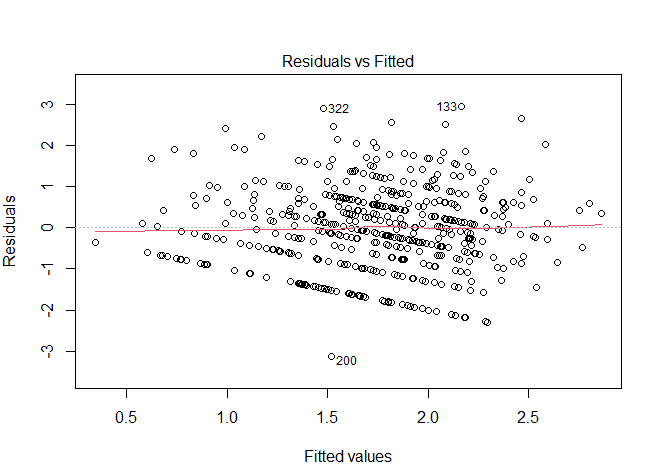

Supplement: online supplemental file 2 [file bmjopen-15-9-s002.docx]
