# Supplementary material for: Association between informal help and background factors for persons with multiple sclerosis in Sweden: a cross-sectional study
Source: BMJ Open. 2025 Sep 14;15(9):e094418. doi: 10.1136/bmjopen-2024-094418 (PMC12434731; doi:10.1136/bmjopen-2024-094418)
Supplement: online supplemental file 3 [file bmjopen-15-9-s003.docx]

**Supplementary file 3 – Non-response rates**

**Supplementary table 3 - Non-response rates reported by Statistics Sweden**

| **Sex** n (%)  Female  Male | 2734 (46.4)  1323 (51.1) |
| --- | --- |
| **Age*** n (%)  20-29 years  30-39 years  40-49 years  50-59 years | 530 (57.7)  1495 (51.5)  1730 (43.8)  291 (42.6) |
| **Birth country*** n (%)  Sweden  Other  countries | 3172 (44.9)  874 (58.4) |
| **Disposable income 2019***  n (%)  None  1–124 999  125 000–199 999  200 000–279 999  280 000–369 999  ≥370 000 | 222 (68.7)  612 (61.6)  501 (57.6)  693 (51.8)  836 (46.6)  1182 (37.6) |

***** Please note that the variable outcomes differ from those reported in the article, as the above figures were reported by Statistics Sweden.
