# Supplementary material for: Association between informal help and background factors for persons with multiple sclerosis in Sweden: a cross-sectional study
Source: BMJ Open. 2025 Sep 14;15(9):e094418. doi: 10.1136/bmjopen-2024-094418 (PMC12434731; doi:10.1136/bmjopen-2024-094418)
Supplement: Supplementary file 4 [file bmjopen-2024-094418.reviewer_comments.pdf]

PEER REVIEW HISTORY

BMJ Open publishes all reviews undertaken for accepted manuscripts. Reviewers are asked to complete a checklist review form and are provided with free text boxes to elaborate on their assessment. These free text comments are reproduced below.

ARTICLE DETAILS

Title (Provisional)

Association between informal help and background factors for persons with multiple sclerosis in Sweden: a cross-sectional study

Authors

Stahl, Daniel; Friberg, Emilie

VERSION 1 - REVIEW

**Reviewer**	**1**
**Name**	**Yazdan Panah, Mohammad**
**Affiliation**	**Shahrekord University of Medical Science**
**Date**	**19-May-2025**
**COI**	**None**

Dear Editor,

I have had the opportunity to review the manuscript titled "Association between informal help and background factors for persons with multiple sclerosis in Sweden: a cross-sectional study." This study examines the prevalence and determinants of informal (unpaid) help received by people with multiple sclerosis (PwMS) in Sweden, focusing on sociodemographic and clinical background factors. Overall, the manuscript addresses a relevant and under-studied aspect of MS care with a robust cross-sectional dataset. The analysis identifies key factors – most notably greater disability (higher EDSS scores), being born outside Nordic countries, and being on sickness benefits – that are strongly associated with both the likelihood of using informal help and the number of hours of help received weekly. The topic is important for understanding how care needs are met among PwMS, and the data provided are valuable. However, several areas of the manuscript would benefit from improvement to enhance its clarity, rigor, and contribution to the literature. Below I detail my concerns:

1. Expand on the impact of excluding individuals over 51 years of age.

2. Discuss potential non-response bias due to underrepresentation of certain groups (e.g., men, lower income, immigrants).

3. Clarify that causality cannot be inferred from cross-sectional data.

4. State whether comorbid conditions or MS disease duration/subtype were analyzed.

5. Interpret the interaction effect (education × sickness benefit) in the logistic model.

6. Report checks for multicollinearity and linear regression assumptions.

7. Clarify sample sizes for regression models and how missing data were handled.

8. Acknowledge low R^2 in linear regression and discuss unmeasured variables.

9. Highlight differences in predictors between 'use of help' and 'amount of help' models.

10. Clearly state novelty in Introduction and emphasize unique contributions.

11. Proofread manuscript for minor typos (e.g., 'Strenghts' → 'Strengths').

12. Focus results narrative on most important findings; refer to tables for full stats.

13. Use subheadings to separate results for 'use of help' and 'amount of help'.

14. Explain interaction terms and include them in tables if not already present.

15. Add literature on prevalence of informal care in MS to Introduction.

16. Compare findings with international studies or sociological research on caregiving.

17. Emphasize practical/policy implications in the Discussion and Conclusion.

18. Suggest future research directions (e.g., longitudinal or qualitative studies).

**Reviewer**	**2**
**Name**	**Morris-bankole, H**
**Affiliation**	**University of Reading**
**Date**	**08-Jun-2025**
**COI**	**None**

I thank the authors for the opportunity to review this novel piece of research. This research was thoroughly researched and performed on a large sample. The methods are clearly outlined from beginning to end.

There are only a couple of points that I feel require further attention at this point.

Firstly, relating to the non responders. I recognise that you acknowledge this as a limitation, but there doesn't seem to be any recognition of how this could have held an impact on the results, especially given that it was noted that there were a higher proportion of non responders who were younger, male, were born outside Sweden, and had a lower income than those who responded, yet the latter 2 demographics were found to be amongst those

more likely to utilise informal caregiving amoungst the responders. Could lack of response from these 2 demographics in particular have had any impact on the study findings? Might there have been some reason why those born outside Sweden and on a lower income were less likely to respond and might there be a relationship between this and accessing informal care?

My second point regards the variable relating to whether responds were born inside or outside of Nordic countries. I'm wondering if data was collected specifically where respondents were born? Given the huge variation in cultural norms regarding informal care globally, this could be a significant contributing factor here worthy of mention in your manuscript.

VERSION 1 - AUTHOR RESPONSE

Reviewer: 1

Mr. Mohammad Yazdan Panah, Shahrekord University of Medical Science

1. Expand on the impact of excluding individuals over 51 years of age.

Authors: We have expanded this discussion in the strengths and limitations section:

"The sample consisted of persons with MS between 20 and 51 years old, due to being collected primarily to focus on younger persons with MS of working age. On the one hand, the young sample is a strength, as previous studies are often based on older samples [e.g. 10]. On the other hand, inclusion of older persons with MS would probably have led to a greater discrepancy between impairment levels within the sample."

2. Discuss potential non-response bias due to underrepresentation of certain groups (e.g., men, lower income, immigrants).

Authors: Thank you for this comment. We have now added more discussion on non-respondents in the strengths and limitations section:

"The proportions of non-respondents were higher in certain groups, which is common in survey-based research [e.g. 47, 48, 49]. This may have been due to a number of reasons, such as the survey being too extensive, the content being perceived as irrelevant [47] or language being a barrier for those born in other countries [47, 48]. It is conceivable that these factors may have influenced the response rate among those invited to participate. Another factor that may be involved is socio-cultural differences in what is perceived to be the responsibility of relatives in helping a sick loved one [cf. 13]. Such differences could be based on whether the

persons were born in a country with a less general welfare system than Sweden [cf. 25]. We cannot know the extent of use of informal help among non-responders, but the interpretation of the extent of informal help in these groups should be made with caution."
The following references that previously were not in the manuscript have been added to support the discussion: Ahlmark et al. (2015), Andersson (2024), Vo et al. (2023) and Katsavos et al. (2017).

3. Clarify that causality cannot be inferred from cross-sectional data.
Authors: We have clarified this in the strengths and limitations bullet points and in the section highlighting the strengths and limitations of the study, following the discussion: "Causality cannot be demonstrated based on cross-sectional data, and the direction of the associations have to be assumed based on what is known from previous research [36]."

4. State whether comorbid conditions or MS disease duration/subtype were analyzed.
Authors: As we were interested in the level of impairment and its possible impact on the use of informal help, we chose to use the EDSS as it is the most widely accepted instrument (cf. Meyer-Moock et al., 2014; van Munster & Uitdehaag, 2017). Regarding comorbidity, the regression analyses included a binary variable involving the possible presence of other long-term disease/impairment. It was found to have a statistically significant association with the use of informal help ($p < 0.05$, OR 1.25) but not with the number of hours of informal help used per week. However, we did not analyze possible differences between other long-term diseases/impairments between respondents, which is a good suggestion for future research that we have added. Thank you.

5. Interpret the interaction effect (education × sickness benefit) in the logistic model.
Authors: We have added an interpretation of the interaction effect between reception of sickness benefit and educational level to the results section:
"The statistically significant interaction effect found between reception of sickness benefit and educational level (see Methods) was included in the model. This interaction indicates that the positive association between receiving sickness benefit and use of informal help is **weaker among those with higher education**."

6. Report checks for multicollinearity and linear regression assumptions.
Authors: We have added two supplementary files. Supplementary file 1 reports Variance Inflation Factor and Tolerance values for the regression analyses. Supplementary file 2 reports assumptions for linear regression, using a PP Plot and residual plot. In the methods section of the manuscript where these checks are referred to, we added references to the supplementary files.

7. Clarify sample sizes for regression models and how missing data were handled.
Authors: We have clarified the sample sizes for the regression analyses in the methods section. In the section where we describe our variables, we have described the missing indicator method (Pedersen et al., 2017) used to handle missing data.

8. Acknowledge low R^2 in linear regression and discuss unmeasured variables.
Authors: We have used this comment to strengthen the part where we suggest directions for future research, thank you.

9. Highlight differences in predictors between 'use of help' and 'amount of help' models.
Authors: We have expanded the discussion regarding differences between statistically significant variables and outcomes from the regression analyses:
"There were also some differences between the results of the logistic regression and the linear regression, worthy of attention, for example regarding age. The OR for using informal help were found to be lower only for the 41–51 age group compared to the reference group. In contrast, the linear regression analysis revealed only a statistically significant difference between the 31–40 age group and the reference group, in the form of a negative relationship. Another interesting difference was that the cohabitation variable was only statistically significant in the linear regression analysis, with an expected negative relationship [cf. 6, 7, 19] to the dependent variable."

10. Clearly state novelty in Introduction and emphasize unique contributions.
Authors: Thank you. We have revised parts of the Introduction, including the part preceding the aim, to more clearly emphasize the contribution of the study:
"To summarize, the state of knowledge about persons with MS and informal help is limited. This is because many studies are based on relatively small samples [e.g. 13], samples with a relatively high average age [e.g. 10] and/or a high degree of impairment [e.g. 14]. There are few Swedish studies in this area, despite the described development towards de-universalization. Previous research has shown that various sociodemographic background factors can influence whether persons in need of help due to disease or age use informal help."

11. Proofread manuscript for minor typos (e.g., 'Strenghts' → 'Strengths').
Authors: Thank you for drawing our attention to spelling mistakes. We have now gone through the manuscript again.

12. Focus results narrative on most important findings; refer to tables for full stats.
Authors: The results section has been revised in line with this and other comments, referring to tables for full stats.

13. Use subheadings to separate results for 'use of help' and 'amount of help'.
Authors: We have added subheadings that separate the reporting of results for the use of informal help and the number of hours of informal help used per week.

14. Explain interaction terms and include them in tables if not already present.
Authors: The found interaction between reception of sickness benefit and educational level is interpreted in the results section and reported in Table 2.

15. Add literature on prevalence of informal care in MS to Introduction.

Authors: We have now added text describing the prevalence of informal help based on the following added references: Katsavos et al. (2017), Kobelt et al. (2017), Maguire & Maguire (2020), and Svensson & Fajutrao (2014).
"One study involving persons with MS from several European countries found that around 46% of respondents used informal help from family members [8]. This figure is broadly consistent with other estimates, although data on this is lacking from various national contexts [12]. A smaller study from Greece reported that around 58% of respondents used informal help [13]. In a Swedish study that primarily examined the societal costs related to both formal and informal help, it was found that 49% of the respondents used informal help to some extent [14]."

16. Compare findings with international studies or sociological research on caregiving.
Authors: Thank you for this comment. The discussion has been revised and expanded in line with this. We have primarily sought to strengthen the connection between the study's results and other research, both Swedish and international. In addition to references that have been added to previous parts of the article (e.g. the Introduction) that are discussed, the following have been added: Ciampi et al. (2023), Price et al. (2020), Rauch et al. (2018), and Rhodes et al. (2008).

17. Emphasize practical/policy implications in the Discussion and Conclusion.
Authors: We have adressed policy implications in the discussion (the last paragraph before Suggestions for future research) and at the end of the conclusions.

18. Suggest future research directions (e.g., longitudinal or qualitative studies).
Authors: Thank you. We have revised this section and added a subheading to it:
"This study can suggest several directions for future research. Although we included the presence of co-morbidity, future studies could explore the issue in more depth by, for example, more specifically analyzing possible differences between types of diseases/impairments. The low adjusted R square value obtained from the linear regression analysis indicates that future studies should further investigate which variables have an impact on the amount of informal help used among persons with MS. It is suggested that

future research should further explore differences in the use of informal help between persons with MS who are domestically born and those born in countries with other types of welfare systems. Furthermore, there is a need for longitudinal studies that would be able to outline any changes in variables associated with the use of informal help over time, but also contribute knowledge on any plausible changes in line with de-universalization [22]. It can also be suggested that qualitative research might be able to explore how persons with MS experience and relate to a situation where they are in need of help. Is the degree of impairment the most influential factor when persons with MS themselves describe their path towards seeking help?"

Reviewer: 2

Dr. H Morris-bankole, University of Reading

- Firstly, relating to the non responders. I recognise that you acknowledge this as a limitation, but there doesn't seem to be any recognition of how this could have held an impact on the results, especially given that it was noted that there were a higher proportion of non responders who were younger, male, were born outside Sweden, and had a lower income thanthose who responded, yet the latter 2 demographics were found to be amongst those more likely to utilise informal caregiving amoungst the responders. Could lack of response from these 2 demographics in particular have had any impact on the study findings? Might there have been some reason why those born outside Sweden and on a lower income were less likely to respond and might there be a relationship between this and accessing informal care?

Authors: We thank you and reviewer 1 for bringing this to our attention. We have now added more discussion on non-respondents in the strengths and limitations section:

"The proportions of non-respondents were higher in certain groups, which is common in survey-based research [e.g. 47, 48, 49]. This may have been due to a number of reasons, such as the survey being too extensive, the content being perceived as irrelevant [47] or language being a barrier for those born in other countries [47, 48]. It is conceivable that these factors may have influenced the response rate among those invited to participate. Another factor that may be involved is socio-cultural differences in what is perceived to be the responsibility of relatives in helping a sick loved one [cf. 13]. Such differences could be based on whether the persons were born in a country with a less general welfare system than Sweden [cf. 25]. We

cannot know the extent of use of informal help among non-responders, but the interpretation of the extent of informal help in these groups should be made with caution."
The following references that previously were not in the manuscript have been added to support the discussion: Ahlmark et al. (2015), Andersson (2024), Vo et al. (2023) and Katsavos et al. (2017).

- My second point regards the variable relating to whether responds were born inside or outside of Nordic countries. I'm wondering if data was collected specifically where respondents were born? Given the huge variation in cultural norms regarding informal care globally, this could be a significant contributing factor here worthy of mention in your manuscript.

Authors: Thank you for a relevant question and reflection. We are aware that ethnicity and/or cultural differences are not easy to measure. In the register data we received from Statistics Sweden, there was information on whether the respondent was born in Sweden, the rest of the Nordic countries, the rest of Europe or the rest of the world, broken down by continent (e.g. North America or Africa). Since a very large majority (n = 3886) were born in Sweden, we chose to merge those born in Sweden with those born in the other Nordic countries, given that these countries have relatively unique, and equivalent, universal welfare systems (Arts & Gelissen, 2010). We are of course aware that this approach is not flawless, but chose to do so given our data. In the section on strengths and limitations, we have expanded on this:
"Lastly, various variables were recoded before being included in the analyses. For example, birth country was coded as a binary variable based on the fact that a large majority of respondents were born in Sweden. Because of this, several other outcomes (e.g. Africa) included very few observations. Based on the fact that the Nordic countries are usually classified as universal welfare states [41], these countries were therefore merged and compared against the others. Statistically, these measures were necessary. However, these actions meant that it was not always possible to reflect the complex reality of the respondents' situation."
We have previously addressed socio-cultural differences as a possible influencing factor in the strengths and limitations.

VERSION 2 - REVIEW

**Reviewer**	**1**
**Name**	**Yazdan Panah, Mohammad**
**Affiliation**	**Shahrekord University of Medical Science**
**Date**	**24-Jul-2025**
**COI**	

Dear Editor,

Thank you for the opportunity to re-evaluate the revised manuscript entitled "Association between informal help and background factors for persons with multiple sclerosis in Sweden: a cross-sectional study." I appreciate the authors' thoughtful and comprehensive responses to my earlier comments. I commend their efforts in improving the manuscript. Below is some additional remarks for further refinement:

1. The manuscript uses EDSS ranges (0–2.5, 3–5.5, 6–9.5). Please briefly justify this categorization in the Methods, especially as the middle category (3–5.5) was not significantly associated with hours of help, unlike the highest category. Clarifying whether this grouping was based on clinical relevance or sample size considerations would enhance transparency.

2. The manuscript alternates between "informal help" and "unpaid help" (e.g., in survey item wording). Although the choice to reflect the survey language is appropriate, a brief clarification of terminology in the Methods section would benefit readers.

3. While the authors address non-response bias in detail, it may be helpful to include response rates disaggregated by key demographics in supplementary material. This would give more precision to the discussion.

4. There is a minor inconsistency in the interpretation of age in the logistic vs. linear models. A sentence reconciling the fact that 31–40 is significant in one model and 41–51 in the other may help readers interpret this more smoothly.

5. The final paragraph of the Discussion ends on a somewhat speculative tone. It may help to briefly restate one or two empirically grounded implications before transitioning to the future research section.

VERSION 2 - AUTHOR RESPONSE

Reviewer: 1

Mr. Mohammad Yazdan Panah, Shahrekord University of Medical Science

1. The manuscript uses EDSS ranges (0–2.5, 3–5.5, 6–9.5). Please briefly justify this categorization in the Methods, especially as the middle category (3–5.5) was not significantly associated with hours of help, unlike the highest category. Clarifying whether this grouping was based on clinical relevance or sample size considerations would enhance transparency.

Authors: Thank you for this comment. This categorization is based on a clinical division within the EDSS scale (e.g., Truong et al., 2021), where the boundary between minimal disability and moderate disability is at the step from 2.5 to 3, i.e., 3 indicates moderate disability. Similarly, a boundary was set between 5.5 and 6, with 6 indicating dependence on aids for mobility. In this way, groups categorized as mild MS (0–2.5), moderate MS (3–5.5), and severe MS (6–9.5) were used. This division has been used in several published studies on the same data (e.g. Ståhl & Friberg, 2023; Machado et al., 2024; Teni et al., 2025). We have clarified this in the manuscript with the following text:

"The categorization of the EDSS variable was based on clinical thresholds [30], where the group between 0–2.5 corresponds to mild MS, 3–5.5 corresponds to moderate MS, and 6–9.5 corresponds to severe MS."

The following reference that previously was not in the manuscript have been added: Truong et al. (2021).

2. The manuscript alternates between “informal help” and “unpaid help” (e.g., in survey item wording). Although the choice to reflect the survey language is appropriate, a brief clarification of terminology in the Methods section would benefit readers.

Authors: It is correct that the term "unpaid help" is used in a few places in the article. In the abstract, we have used it to explain simply and directly what the concept of "informal help" means. In the Methods section, under the subheading Data, we clarify that the term "unpaid help" rather than "informal help" was used in the survey question. This is because "unpaid help" is a more colloquial term, and we wanted to avoid potential respondents not understanding what the question was about. In research, however, it is customary to use "informal", although there may be some variation in whether "help" (e.g., Dahlberg et al., 2018) or "care" (e.g., Zhang & Sun, 2020) is used. Out of respect for the movements of disabled people, we have chosen to use the term "help" (cf. Shakespeare, 2000; Jeppsson Grassman et al., 2009), thus "informal help". We have reviewed the article to ensure that "unpaid help" is not used unnecessarily, thank you for your comment.

3. While the authors address non-response bias in detail, it may be helpful to include response rates disaggregated by key demographics in supplementary material. This would give more precision to the discussion.

Authors: Thank you for this suggestion. We have added a Supplementary file 3 where Supplementary table 3 shows the non-response rates for the variables in question. Please note that these figures were reported by Statistics Sweden and that the outcomes therefore differ in several cases from those in the article. We have added a reference to Supplementary file 3 in the article's Strengths and limitations section.

4. There is a minor inconsistency in the interpretation of age in the logistic vs. linear models. A sentence reconciling the fact that 31–40 is significant in one model and 41–51 in the other may help readers interpret this more smoothly.

Authors: Thank you for noticing this. We have revised to be more precise into the following: "The logistic regression analysis showed a statistically significant lower OR for the use of informal help for the group aged 41-51 compared to the reference group, while the linear regression analysis showed a statistically significant negative relationship with the number of hours of informal help used per week for the 31-40 age group compared to the reference group."

5. The final paragraph of the Discussion ends on a somewhat speculative tone. It may help to briefly restate one or two empirically grounded implications before transitioning to the future research section.

Authors: In the final paragraph of the Discussion, we have briefly referred back to the results of this study as well as our previous one, based on the same data:

"The often invisible impact of the disease was also reflected in this study. In terms of the proportion of persons with MS who used informal help, those who reported an invisible symptom as the most limiting accounted for the substantially largest group. In our previous study, we found that visible symptoms were related to the use of personal assistance, while invisible symptoms were related to the less extensive help form of home help services [39]."

We have also added a sentence at the end of the paragraph that ties the paragraph together:

"This suggests that invisible symptoms need to be given greater attention within healthcare and by the authorities that decide on formal help."
To make the transition in the next paragraph a little smoother, we have added the following sentence:
"In line with what was discussed in the previous paragraph, we suggest that future studies further investigate the impact of invisible and fluctuating symptoms of MS."
We thank you for the suggestion.
